# Nickel and cadmium-induced SLBP depletion: A potential pathway to metal mediated cellular transformation

**DOI:** 10.1371/journal.pone.0173624

**Published:** 2017-03-17

**Authors:** Ashley Jordan, Xiaoru Zhang, Jinquan Li, Freda Laulicht-Glick, Hong Sun, Max Costa

**Affiliations:** 1 Department of Environmental Medicine, New York University School of Medicine, Tuxedo, New York, United States of America; 2 Department of Biochemistry and Molecular Pharmacology, New York University School of Medicine, New York, NY, United States of America; 3 Hubei Key Laboratory of Genetic Regulation and Integrative Biology, School of Life Science, Central China Normal University, Wuhan, Hubei, China; Augusta University, UNITED STATES

## Abstract

Both nickel and cadmium compounds have been established as group I carcinogens for several decades. Despite over-whelming evidence of these compounds’ carcinogenicity in humans, the specific underlying molecular mechanisms that govern metal induced cellular transformation remain unclear. In this study, we found that there were slightly different effects on decreased SLBP mRNA and protein as well as increased polyA H3.1 in our nickel exposed cells. This suggested that nickel and arsenic have similar effects on canonical histone mRNA transcription and translation. We also saw that the depletion of SLBP protein was reversed by inhibiting the proteosome. Finally, we showed that inhibiting the SLBP mRNA and protein levels were rescued by epigenetic modifiers suggesting that nickel’s effects on SLBP may be mediated via epigenetic mechanisms. Taken together these results suggest a similar mechanism by which both arsenic and nickel may exert their carcinogenic effects.

## Introduction

Both nickel and cadmium are well-established carcinogenic metals with historical evidence of human exposure associated with increased incidences of various cancers reviewed and studied here [1–5]. Nickel compounds were classified as group I carcinogens in 1970, with the most dangerous forms of nickel compound exposure occurring occupationally [6]. Cadmium and cadmium compounds were classified as carcinogen later also, due to over-whelming correlative evidence between cadmium exposure [7]. Vulnerable individuals include those who are regularly exposed to toxic metal dust of heterogeneous composition (i.e. soluble and insoluble nickel and cadmium compounds)—usually during processes such as electroplating, smelting, mining nickel ores, or battery manufacturing[3, 8–11]. Despite indications of its carcinogenic properties, nickel has been shown to be a weak mutagen [12–14]. Nickel has also been shown to induce cellular transformation in *vitro* and induce gene expression changes in the circulating PBMCs of nickel refinery workers as compared to controls [15–17]. Numerous studies indicate that nickel's adverse health effects are likely mediated by epigenetic changes [16–19]. More specifically, nickel has been shown to increase chromatin condensation via increased DNA methylation and decreased histone acetylation this, in turn, facilitates significant gene expression changes *in vitro and in vivo* [16, 19–23]. Despite many studies of nickel's effects, a clear mechanism by which nickel induced cellular transformation and carcinogenesis occurs remains unclear. Investigating these pathways could yield greater insight into nickel induced carcinogenesis and potential therapeutic interventions for those at higher risk for nickel related respiratory diseases and lung cancers. Similarly, the molecular effects of cadmium include inhibition of DNA repair, gene silencing, increased stress pathway response, and reactive oxygen species. Like nickel, cadmium exposure correlates with several cancers including lung, breast, and prostate. Cadmium has been shown to modify epigenetic mark such as histone methylation, but a clear pathway to cellular transformation has yet to be identified.

Our lab has previously shown that arsenic, another toxic/carcinogenic metal, induces the inappropriate expression of canonical/replication dependent histone H3.1 through depletion of stem loop binding protein (SLBP) and its subsequent poly-adenylated mRNA [21, 22]. Canonical histone mRNAs (H2A, H2B, H3, and H4) are unique in that they do not end in a typical poly–adenylation (polyA) sequence. Instead, canonical histone mRNAs end in a conserved stem loop structure to which SLBP binds and facilities strict temporal trafficking, translation and stability [24–26]. SLBP protein expression is tightly coupled with the cell cycle and begins to accumulate at the G1/S border. Protein levels stay high during S phase and rapidly decrease at the end of S phase [27–30]. We found that in the absence of SLBP the default mechanism of polyadenylation occurs and canonical histone mRNAs are transcribed with a polyA tail. Because histone mRNAs in cells with metal-induced SLBP depletion were polyadenylated at a higher rate, we hypothesized that they become more stable finally leading to higher translation and inappropriate incorporation into the genome [22, 23]. Yet another consequence of polyA histone mRNAs includes the uncoupling of histone mRNA degradation from the cell cycle because SLBP cannot act as a chaperone. This uncoupling also increases the potential for more translation of histone protein. In this study, we explore a novel pathway by which nickel and cadmium may exert their carcinogenic effects via SLBP depletion and increased histone h3.1mRNA expression and stability.

## Materials and methods

### Cell culture and metal exposures

Beas2B and BL41 cells were grown in DMEM or RPMI media respectively at 37°C and 5% CO2. All media was supplemented with 10% bovine serum and 1% penicillin/streptomycin. Cells were grown to approximately 70–80% confluence and sub-cultured to maintain optimal growth. For nickel exposures, cells were plated at 40% confluency and allowed to grow in fresh media for 24 hours. After 24 hours, nickel chloride (Ni Cl_2_) or cadmium chloride (Cd Cl_2_) was added to the media at final concentrations of 0μM, 100 μM, 250μM or 400μM Ni Cl_2_ or 0μM, 1μM, 2.5 μM, 5 μM Cd Cl_2_.Cells were allowed to grow in metal containing media for 48 hours before collection and extraction of whole cell lysates or total RNA for further analysis. For co-treatment experiments, cells were exposed to nickel with or without 10 μM of the proteosome inhibitor MG132, 5 mM of histone deacetylase (HDAC) inhibitor sodium butyrate (Na-Buty), or 10μM of DNA methylation inhibitor 5–AzaCytidine (5AzC). MG132 was added to nickel containing media 1 hour prior to collection, Na-Buty was added 24 hours after the initial nickel exposure and 5 AzC was added at the same time as nickel and kept in the media for 48 hrs.

### RNA isolation and qPCR analysis

Cadmium and nickel exposed cells were collected in trizol and either stored at -80°C or processed immediately for RNA isolation. A hybrid trizol–RNAeasy (Qiagen) protocol was used to isolate RNA. Briefly, 200μl of chloroform was added / 1 ml of trizol used, samples were shaken by hand for 30 seconds and allowed to sit at RT for 2 minutes. Samples were then centrifuged at maximum speed for 18 minutes after which the clear organic layer was added to a clean tube. An equivalent volume of 100% EtOH was added drop-wise, gently mixed, and the total mix was added to RNAeasy columns after which point the manufactures protocol was used to isolate RNA. Total RNA was quantified on the nanodrop. Following quantification, 250 – 500ng of RNA was used to synthesize cDNA using the Superscript III RT-qPCR kit (Invitrogen). Both total and poly(A) pools of cDNA were generated and subsequently analyzed for quantitative amounts of SLBP, histone H3.1, and or gamma tubulin. Primer sequences for all specified genes are included in Table 1

**Table 1:** Primer Sequences. The sequences for all primes used for qPCR experiments are listed. Gamma Tubulin was used as an endogenous control.

Gene	Forward	Reverse
Histone H3.1 (H3.1)	ACGCCAAGCGGGTGACTAT	TCTCGCCGCGGATACG
Stem Loop Binding Protein (SLBP)	CGGCTGAATGACAGGTATCCTA	CTCGTCCTGGTTGGGAAACA
Gamma Tubulin (γTub)	GGGATGGTGCGGATCTACAG	GTTGACTGGGTTTGTATCCTGAAGA

### Whole cell protein extraction

Cells were exposed to the conditions described and collected at the indicated time-points. Cells were then lysed and/or spun, washed in 1X cold PBS and incubated in hot boiling buffer at approximately 95°C for 5 minutes. Samples were then sonicated at maximum speed for 10 minutes in a 4°C water bath and finally centrifuged at maximum speed for 15 minutes. Supernatants were collected and used for subsequent Western blot analysis. Approximately 10–20 μg of protein were loaded onto a precast SDS-PAGE gel with a 4–15% gradient (BioRad) at 100 V for 2 hours. Proteins were transferred to a PVDF membrane at 50 V overnight. Membranes were then blocked in 5% non-fat milk in TBS-T for one hour and incubated with SLBP primary antibody (Abcam) at a concentration of 1:1000 or Tubulin (Abcam) at 1:3000 overnight. Finally, membranes were incubated in goat anti-rabbit HRP conjugated secondary antibody at 1:15000 for 1 hour. Protein bands were detected using ECL detection reagents (Pierce).

### Image J analysis

To quantify the Western blot images, the Image J software Western blot protocol was used as previously described. Briefly, Western blot images were converted to 8-bit Jpeg images and the intensity of SLBP bands were quantified using tubulin as a loading control with densitometry. Calculations for band intensity were computed using Microsoft Excel.

### Significance testing

Statistical significance was calculated using the online Graph Pad software unpaired, 2-tail t-test. For p-values < 0.05 * is used as an indicator and for p-values < 0.01 ** is used as an indicator.

## Results and discussion

### Stem-loop binding protein (SLBP) is depleted in nickel chloride (Ni Cl_2_) and cadmium chloride (Cd Cl_2_) exposed cells

**Fig 1:**
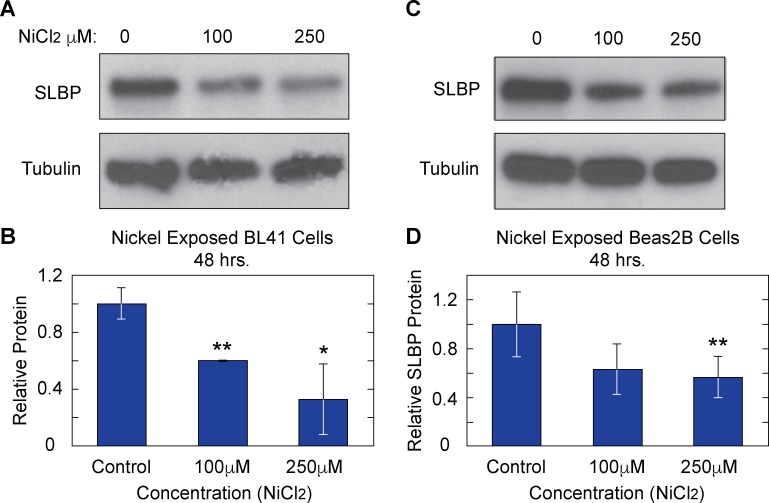
Nickel exposure depletes SLBP protein in Beas2B and BL41 cells. Whole cell lysates from BL41 (A) and Beas2B (C) cells exposed to the Ni Cl_2_ for 48 hours show marked decreases in SLBP in representative western blots. Relative protein amounts for were quantified and shown in charts (B) and (D).

**Fig 2:**
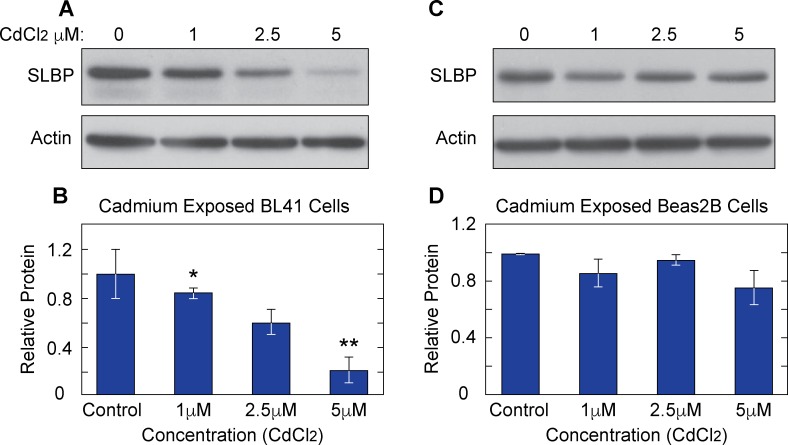
Cadmium exposure depletes SLBP in Beas2B cells and BL41 cells. Cell lysates from BL41 cells exposed to 0μM, 1μM, 2.5μM and 5 μM Cd Cl_2_ 24 hrs (A) and Beas2B cells exposed for 48 hrs (C) were analyzed via western blot. Protein levels were quantified and shown in graphs B and D

In order to recapitulate conditions under which nickel induces cellular transformation and gene expression changes occur, both Burkitt’s lymphoma cells (BL41) and normal human bronchial epithelial lung (Beas2B) cells were treated for 24 or 48 hrs with 100 μM or 250 μM nickel chloride (Ni Cl_2_). Whole cell lysates were collected in boiling buffer from nickel exposed and control samples. Western blot analysis showed a clear decrease of SLBP protein levels in BL41 (Fig 1A and 1B) and Beas2B (Fig 1C and 1D). There were decreases of approximately 35% and 45% in SLBP at 100 μM and 250 μM of Ni Cl_2_ respectively in BL41 cells and decreases of approximately 50% and 45% in SLBP protein at 100 μM and 250 μM in Beas2B cells. It should be noted that these doses of nickel were not toxic to the cells during the time period of exposure but higher doses that were used (400 μM and 500 μM) did show slight toxicity (data not shown). Cadmium is also an established carcinogenic metal known to induce epigenetic changes and has been thoroughly reviewed here [28, 29]. In order to determine if SLBP depletion occurs in cadmium exposed cells as well, BL41 cells were exposed to 0μM, 1μM, 2.5μM, and 5μM Cd Cl_2_ for 24 hours and Beas2B cells were treated with the same concentrations for 48 hours. Fig 2A and 2B show Western blot images of samples collected from each cell type respectively. Densitometry analysis indicated that there were notable decreases of SLBP protein in Cd Cl_2_ exposed BL41 and Beas2B cells respectively. This result suggested that cadmium has a similar effect on SLBP protein levels as nickel and arsenic. An apparent concentration dependent depletion of SLBP protein can be seen in BL41 cells Cd Cl_2_.

**Fig 3:**
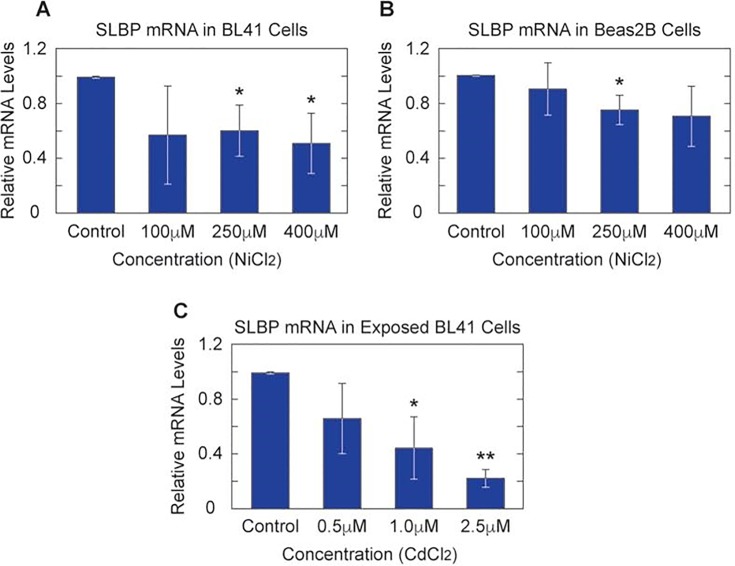
Both nickel and cadmium significantly decrease SLBP mRNA in exposed cells. Total RNA was collected from nickel and cadmium exposed cells and converted to cDNA. RT-qPCR analysis showed clear decreases in SLBP mRNA in Nickel treated BL41(A) and Beas2B cells (B). Cadmium exposed BL41 cells exhibit a similar pattern (C).

Both nickel and cadmium induce changes in gene expression patterns, thus we sought to determine if the underlying mechanism of SLBP protein depletion was due to a decrease in SLBP mRNA gene expression. Total RNA was isolated from nickel and cadmium exposed Beas2B and BL41 cells. Approximately 250 ng of RNA isolated from each exposure sample was used to create cDNA pools for quantitative real-time PCR (RT-qPCR) using primers against SLBP and λ-Tubulin. Nickel induced significant decreases in SLBP mRNA in BL41 and Beas2B cells (Fig 3A and 3C). There was an approximately 40% and 50% decrease in SLBP mRNA in BL41 cells with 250μM and 400μM Ni Cl_2_. Cadmium exposure however, had a less pronounced reduction of SLBP mRNA in Beas2B cells (data not shown) relative to the effect seen in BL41 cells (Fig 3C) with approximately 40%, 60% and 80% decreases with increasing concentrations of Cd Cl_2_.

### The effect of nickel and cadmium exposure is heritable

**Fig 4:**
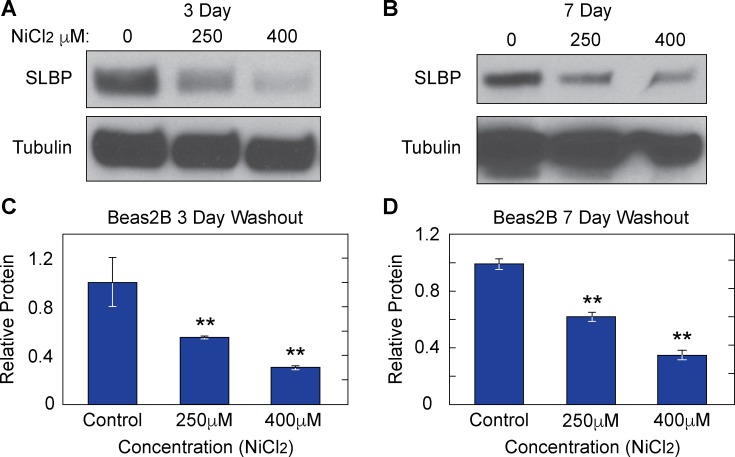
Nickel induces heritable depletion of SLBP protein in Beas2B cells. Beas2B cells were exposed to 0μM, 250μM, or 400μM Ni Cl_2_ for 48 hours. Cells were then washed with PBS and sub-cultured for 3 and 7 days post-exposure. Whole cell lysates were collected and run on a 4–15% gradient SDS PAGE gel. After 3 days (A) and 7 days (B) the decrease in SLBP is still apparent. The relative protein amounts across several experimental replicates are also quantified for both 3 days (C) and 7days (D) post nickel exposure.

**Fig 5:**
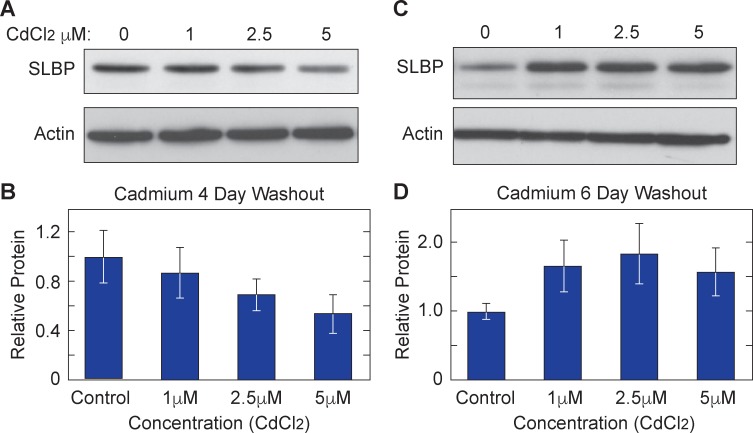
Cadmium induces heritable depletion of SLBP protein in BL41 cells. BL41 cells were exposed to 0μM, 1μM, 2.5μM, or 5μM Ni Cl_2_ for 48 hours. After removing the media and washing in PBS, whole cell lysates were collected and run on a 12% SDS PAGE gel after 4 days (A) and 6 days (C). While a decrease in SLBP remains after 4 days, by day 6 SLBP levels are restored to above baseline. The relative protein amounts are quantified for 4 days (B) and 6 days (D).

**Fig 6:**
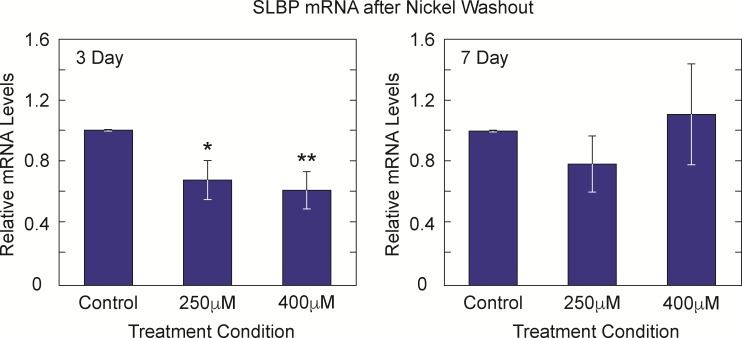
Heritable decreases in SLBP mRNA remain 3 and 7 days post nickel exposure. Total RNA was collected from Beas2B cells that were exposed to nickel and then sub-cultured in fresh media for 3 and 7 days. Significant decreases in SLBP mRNA are seen 3 days post nickel exposure. The same trend holds true for 250μM Ni Cl_2_ after 7 days although the decrease is not significant however, at 400μM Ni Cl_2_ SLBP mRNA levels are restored.

Nickel and cadmium are both well-established epigenetic modifiers [4, 17–19, 30]. One of the hallmarks of ‘epigenetic change’ includes persistent changes in gene expression that are inherited and therefore last several generations after the initial toxin/ carcinogen is removed (*in vitro* and *in vivo)* from the immediate area. Because there was a striking decrease in SLBP protein levels with Ni Cl_2_ and Cd Cl_2_, we wanted to determine how long that change persisted after the initial exposure to these metals had ended. After initial exposure, Beas2B cells were washed with PBS and sub-cultured for 3 or 7 days in the absence of the metal. Whole cell lysate analysis of western blots showed that SLBP protein levels remained low at day 3 (Fig 4A) and day 7 (Fig 4B). Densitometry was used to quantify protein levels in Beas2B cells at day 3 (Fig 4C) and day 7 (Fig 4D). Although the SLBP protein levels in nickel treated cells recovered somewhat between 3 and 7 days, they were still lower than control cells at each respective time point. Cadmium exposed BL41 cells displayed the same depletion pattern at 4 days (Fig 5A) but by day 6 (Fig 5C) SLBP levels were restored to slightly above control levels. Given that both nickel and cadmium are weak mutagens, it is possible that there is some epigenetic change to the genome that was passed down to subsequent cellular generations. To test whether SLBP mRNA expression remained low after nickel washout, total RNA was isolated from nickel washout samples and RT- qPCR was performed to measure SLBP mRNA levels. Table 1 shows the primer sequences for all Fig 6 shows a dose dependent decrease in SLBP mRNA levels at day 3 post nickel exposure. The same trend was present at 7 days in the 250μM Ni Cl_2_ treated sample but not in the 400μM dose of Ni Cl_2_. The lasting effect of nickel exposure at the transcriptional level further supports the hypothesis that changes to the epigenome are being retained by the cell and passed to subsequent cellular generations. Having established that a significant reduction of SLBP protein and mRNA occur both during and after nickel and cadmium exposure, we investigated downstream molecular events that occur as a result of the loss of SLBP. In arsenic exposed cells, a major consequence of depleted SLBP includes increased amounts of poly(A) canonical histone H3 isoform H3.1. With lower levels of SLBP canonical histone mRNA transcript is improperly processed and acquires a poly (A) tail ‘by default’. Given this previous results, we were interested in determining if this occurs in the context of nickel and cadmium induced SLBP depletion as well.

### Nickel and cadmium alter H3.1 mRNA expression patterns and transcript composition

**Fig 7:**
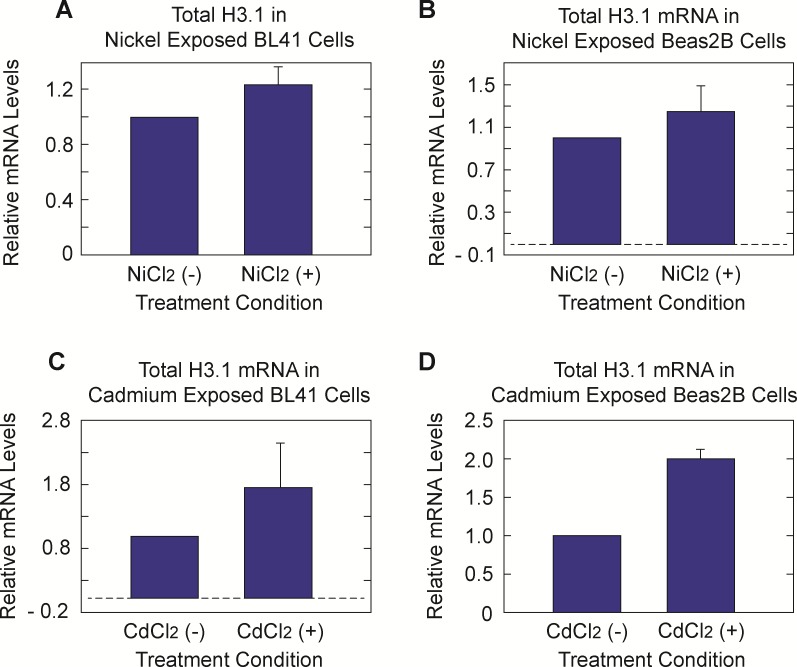
Increase of total H3.1 mRNA in nickel and cadmium exposed cells. Total RNA was isolated from Beas2B and BL41 cells exposed to 0μM or 250μM Ni Cl_2_ and 0μM or 5μM Cd Cl_2_. RT-qPCR was preformed and revealed an increase in the amount of H3.1 mRNA in nickel treated BL41 (A) and Beas2B (B). BL41 (C) and Beas2B (D) showed increased total H3.1 after Cd Cl_2_ also.

**Fig 8:**
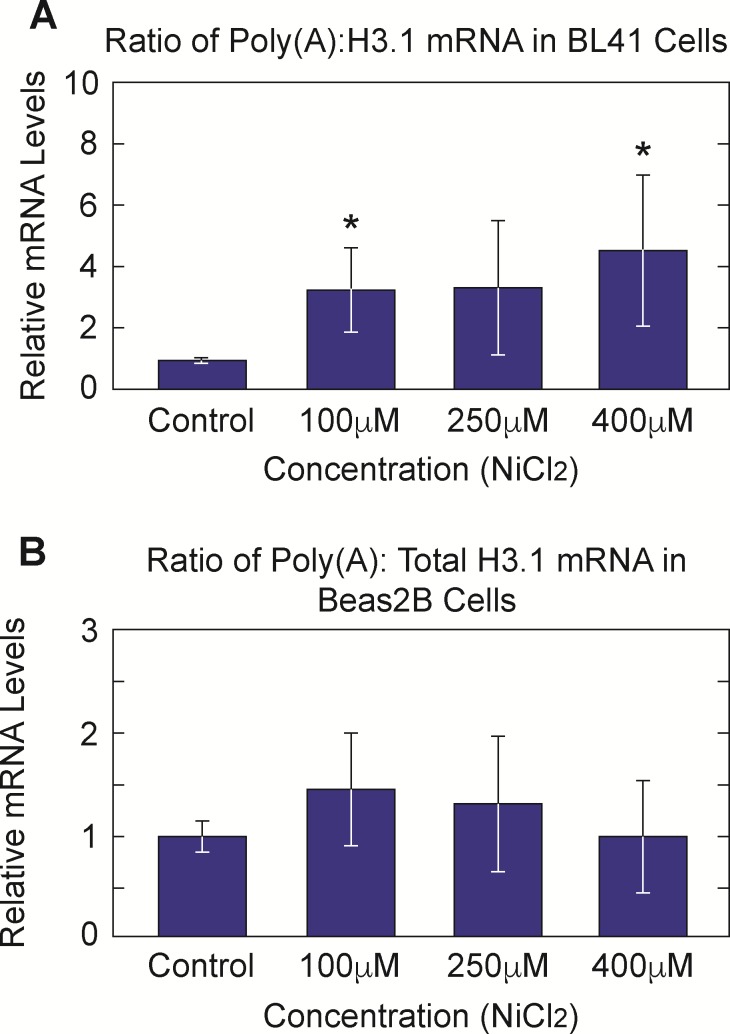
Nickel, but not cadmium, induces an increase in Poly adenylated H3.1 mRNA. Total RNA was collected from Beas2B and BL41 cells treated with 0μM, 100μM, 250μM, and 400μM Ni Cl_2_. During cDNA synthesis, random and olig d(t) primers were used to create pools both total and poly-adenylated cDNA. Real-Time qPCR analysis shows that there is an increase in the amount of poly(A) H3.1 as compared to total H3.1 in nickel treated BL41 (A) and Beas2B (B) cells.

We previously noted a correlation between metal induced depletion of SLBP and an increase in total and poly(A) containing canonical histone H3.1 mRNA [19]. RT-qPCR analysis of nickel and cadmium exposed Beas2B and BL41 cells revealed an increase in total H3.1 levels (Fig 7A–7D). We postulate that this increase is due to an upturn in mRNA stability driven by the partial loss of SLBP and acquisition of an otherwise absent poly(A) tail. Although total H3.1 mRNA increased it was important to determine if ratio of polyA H3.1 mRNA also increased because it is the aberrant addition of the poly (A) tail that appears to drive transformation. Higher amounts of poly(A) histone mRNAs would further support the hypothesis that both nickel and cadmium may have induced cellular transformation via abnormally stable histone mRNAs. Fig 8A and 8B show an increase in the ratio of poly (A) H3.1 mRNA in nickel exposed Beas2B and BL41 cells. We see that there is only a clear dose dependent response in the BL41 cell line and not Beas2B. It is possible that because Beas2B is a ‘normal’ cell line, protective mechanisms against toxic metals are induced to a greater extent thereby mitigating nickel’s effects. Despite this, it is clear that there is an effect of nickel on poly(A) H3.1 in both cell lines. This trend was not evident in cadmium exposed BL41 and Beas2B cells (data not shown). It was at this point that the molecular effects of nickel and cadmium diverge in the context of this investigation. With this in mind, we decided to focus on nickel for subsequent investigation of the mechanism by which SLBP is depleted.

Previous studies have shown that depletion of SLBP by siRNA to less than 5% of its normal cellular level has effects on cell cycle progression (blockage in S phase), cell growth, levels of canonical histone mRNA, and polyadenylation of canonical histone mRNA [25]. With nickel and cadmium exposure in the present study, we only observe a 40–50% depletion of SLBP which may explain some of the differences in results found between the previous study[25] and our findings. Similarly, in our previous study we found that arsenic exposure resulted in no more than a 50% decrease in Beas2B cells but did induce an increase in both H3.1mRNA polyadenylation and total H3 protein [21]. The most striking difference was the reported loss of mRNA for canonical histones in the previous study [25] whereas we observed increases in mRNA for H3.1.This is likely due to the increased polyadenylation of H3.1 resulting in greater stability of the mRNA. In addition, the previous study found that canonical histone mRNA accumulated in the nucleus; this was attributed to the decreased capacity of SLBP to act as canonical histone mRNA chaperone due to very efficient SLBP knockdown (< 5% of basal) [25]. To reiterate, we believe that the differences seen in our findings and those of *Sullivan K*.*D*., *et*.*al* [25] are due to the drastic extent to which SLBP is reduced by metal exposure versus siRNA knock down.

### Perturbations at the transcriptional and translational levels play a role in nickel mediated SLBP depletion

**Fig 9:**
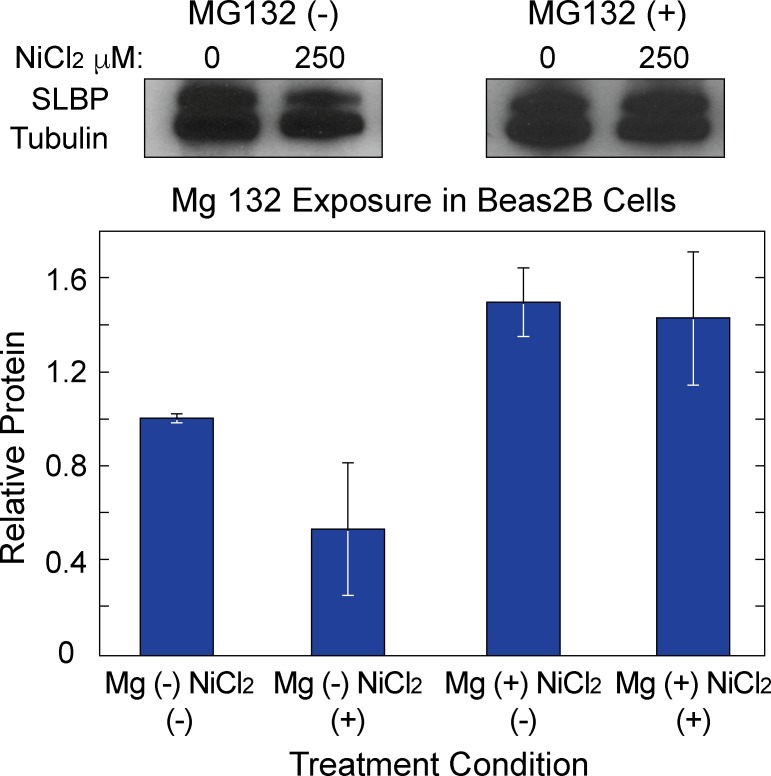
Increased proteosomal degradation plays a role in nickel mediated SLBP protein depletion. Nickel exposed Beas2B cells were co-treated with 10 μM MG-132 for 1 hour before the end of a 48-hour nickel treatment. Whole cell lysates were collected and run on a 4–15% SDS-PAGE gel (A) Densitometry (B) indicated that inhibited proteosomal degradation rescues SLBP protein levels.

**Fig 10:**
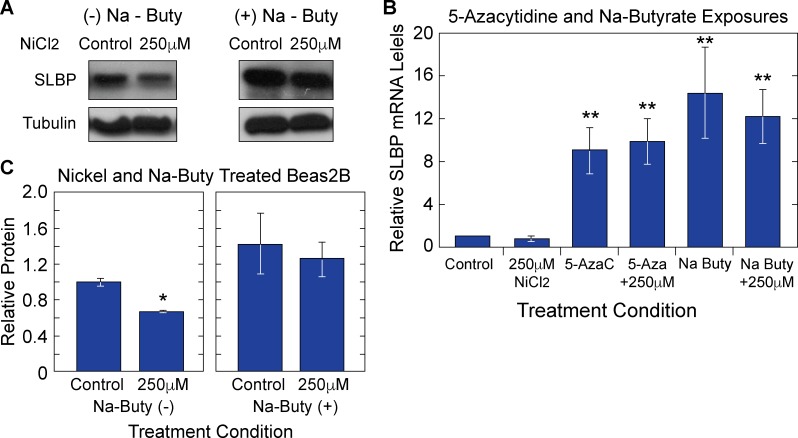
Epigenetic modifiers increase/rescue SLBP protein and mRNA levels in Nickel exposed Beas2B cells. Nickel exposed Beas2B cells were co-treated with Ni Cl_2_ and sodium butyrate (Na–Buty). SDS-PAGE gel (A). Quantification of the western blot indicated that SLBP protein levels are rescued in cells exposed to nickel and sub-cultured with or without Na–Buty (C). Total RNA was collected from cells co-treated with Na–Buty, 5 AzC, or nothing and converted to cDNA. RT-qPCR analysis indicated that SLBP mRNA levels were increased by both epigenetic modifiers (B).

Having established that there are likely heritable SLBP gene expression changes as well as increased levels of total and poly(A) H3.1, we sought to investigate the mechanisms by which nickel mediated SLBP protein and mRNA depletion occurred. To parse the possible post-translational effects of nickel, Beas2B were co-treated with 250 μM nickel and 10 μM MG132. MG132 was added 1 hour before the end of a 48-hour nickel exposure. In the presence of MG132, nickel mediated depletion SLBP protein is mitigated (Fig 9A and 9B) suggesting that increased targeting to the proteasome plays at least a partial role in nickel mediated SLBP protein depletion. To further explore mechanisms by which nickel induces changes in SLBP expression, both transcriptionally and translationally, cells were co-treated with modifiers, sodium butyrate (Na–Buty) or 5 –aza–cytidine (5 AzC) to determine if SLBP depletion could be reversed using either chemical modifier. Our rationale for using 5AzC lies in that nickel has been shown to increase DNA methylation. Because methylation is a silencing mark, it could account for the decreased levels of SLBP mRNA and protein upon nickel treatment [18]. To determine the potential role of increased DNA methylation in SLBP depletion, Beas2B cells were treated with 10uM of 5 AzC during a 48-hour nickel exposure. RT-qPCR analysis revealed a significant increase (Fig 10B) in SLBP mRNA levels in the presence of nickel indicating that DNA methylation changes may be involved in nickel induced SLBP depletion. Nickel has also been shown to inhibit histone acetyltransferase (HAT) activity effectively decreasing histone acetylation in the promoter region of genes [31]. Histone acetylation is an activating epigenetic mark as it promotes an open chromatin structure and increased gene expression [31, 32]. To determine if decreased histone acetylation was important for nickel mediated SLBP depletion, Beas2B cells were initially exposed to nickel for 48 hours and subsequently treated with 5 mM na-buty for an additional 24 hours. After na-buty treatment, both SLBP protein and mRNA levels in nickel treated cells were restored to above control levels (Fig 10A–10C). Taken together, these results suggest that nickel induced depletion of SLBP were caused by aberrant regulation at the transcriptional and post-translational levels.

## Discussion

**Fig 11:**
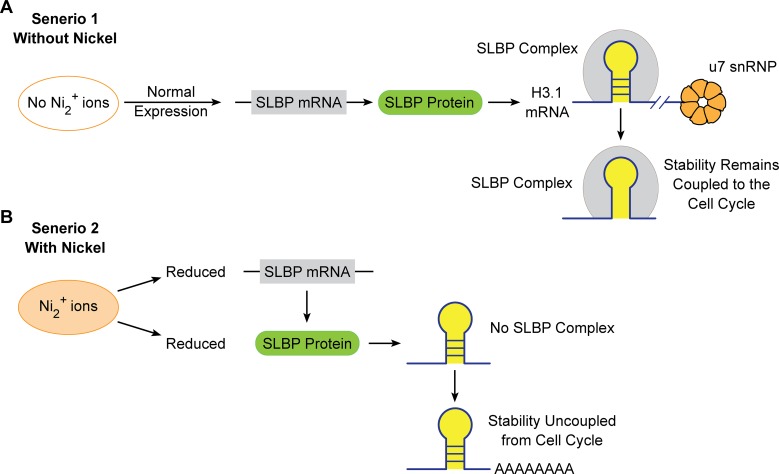
Nickel mediated SLBP depletion increases aberrant H3.1 mRNA polyadenylation. In the absence of nickel, normal SLBP mRNA and protein production allows for normal processing of H3.1 pre-mRNA. Our data indicates that nickel depletes SLBP mRNA and protein levels. This loss prevents the 3’ end cleavage of H3.1 mRNA and the addition of a poly(A) tail which confers increased stability. Increased stability of H3.1 mRNA could potentiate abnormal nucleosome assemble and genomic instability which is deleterious for normal cell growth.

Our lab has previously shown that carcinogenic / toxic metalloid arsenic facilitates an increase in histone mRNA and protein expression presumably through a depletion of SLBP. The inappropriate increase in histone mRNA and subsequent increases in histone protein, resulted in excess histone sub-units that can then be incorporated into chromatin and cause genomic instability. In order to determine if this was a general mechanism by which various other metals exert such effects, we investigated nickel and cadmium for their ability to activate the same pathways. Many studies have established arsenic, nickel, and cadmium as carcinogenic metals but the mechanisms by which they transform cells remains somewhat unclear. Thus, with this investigation, we sought to determine if a common mechanism could be found for several different metals involving depletion of SLBP and increased poly A of canonical histones. Fig 11 depicts a schematic summation of the general mechanism by which nickel may be increasing poly-adenylated canonical histone H3.1 mRNA.

### Nickel and cadmium mediated SLBP depletion occurs in vitro

Our lab is the first to report a decrease in SLBP protein and mRNA levels after nickel and cadmium exposure. We also saw a simultaneous increase in total H3.1 mRNA. These results are significant because our previous report on arsenic indicated that depletion of SLBP, increases in H3.1 mRNA levels (total and poly A) and can potentiate carcinogenic transformation in vitro. Additionally, the effect of both nickel and cadmium can be seen days after removal of the metal ions from cell culture media. This is important because it supports the hypothesis that both metals are introducing heritable changes to the epi-genome that persist ‘generations’ after the initial exposure is removed. It should be noted that nickel, but not cadmium, increased poly(A) H3.1 mRNA suggesting that nickel and arsenic may have similar routes of carcinogenicity. It is likely that nickel decreased SLBP mRNA levels, at least in part, via increased DNA methylation and or decreased histone acetylation in the SLBP promoter region. Previous investigations have indicated that in the absence of SLBP, canonical H3.1 histone mRNA acquires an otherwise absent poly-adenylated tail [21, 22]. This study confirms those findings in that 48 hours of nickel exposure reduces SLBP protein and mRNA levels while simultaneously increasing poly(A) H3.1 mRNA (Figs 3A, 3B, 8A and 8B) in nickel exposed cells. The decreases seen in the protein levels of SLBP appear to be more dramatic that the decreases seen in mRNA levels indicating that the nickel is acting at both the transcriptional and translational levels. With regard to a specific transcriptional effect of nickel, we saw a rescue of SLBP mRNA by histone deacetylase inhibitor Na-buty and DNA methylation inhibitor 5AzC. Nickel decreases acetylation by inhibiting histone acetyltransferase activity, thus it stands to reason that hypo-acetylation at the SLBP promoter could account for decreased transcription and mRNA levels. Our results support this hypothesis in that when Na-buty was used to compensate for the potential imbalance of histone acetylation a dramatic rescue of both SLBP mRNA and protein was observed. Nickel is also a well-established promoter of global DNA methylation most likely due to inhibition of DNA methylation via inhibition of histone demethylase activity [33]. Upon treatment of nickel exposed Beas2B cells with 5 AzC, levels of SLBP mRNA were also dramatically rescued. Taken together, these results along with the lasting SLBP mRNA depletion in Beas2B cells after 48 hours of nickel exposure provide strong evidence that nickel is epigenetically silencing SLBP mRNA. Future experiments will be focused on finding specific histone and DNA modification marks in the promoter region of SLBP that are altered in the presence of nickel. We also sought to delineate if there was a role for proteosomal degradation in nickel mediated SLBP depletion at the protein level. Current data on the specific effects of nickel on proteosomal machinery indicate that nickel may increase proteosomal activity [24]. We saw evidence of this when SLBP protein levels were rescued by MG132. SLBP degradation is partially regulated by cyclin A/Cdk1 phosphorylation on Thr 61 of the SLBP protein at the end of S phase. Ding *et*. *al*. showed that nickel induces a slight accumulation of cells in S phase [31]. Because SLBP is degraded toward the end of S phase it is possible that during this prolonged S phase, SLBP is phosphorylated more frequently and thus degraded at higher rates in nickel exposed cells. It should also be noted that there could be other post translational modifications including sumoylation, acetylation, and ubiquitination that have yet to be characterized for their effect on SLBP degradation in the context of nickel treatment. The modification status of various amino acids in SLBP at known and unknown regulatory sites should be further interrogated upon nickel treatment to determine which if any, play a role in increased SLBP protein degradation.

### The significance of increased polyadenylated H3.1 mRNA

Canonical replication dependent histone expression is tightly linked to the cell cycle and is absolutely required for cell survival. Previous studies have implicated abnormal overexpression of histones in cellular transformation [22]. Our data suggests that nickel exposure facilitates the abnormal stability of at least one canonical histone isoform (H3.1). Because SLBP is the main factor responsible for temporal degradation for canonical histone mRNA, the addition of the polyA tail to H3.1 has the potential to uncouple the degradation of mRNA transcripts from the cell cycle. It is likely that these transcripts remain intact outside of the S phase at which point “normal' canonical histone H3.1 mRNAs would be degraded. This, in turn, could lead to an excess of histone H3.1 proteins that would incorporate into chromatin and confer genomic instability to the exposed cell. Further investigation is needed to confirm if and to what extent these polyA H3.1 mRNAs transcripts remain intact outside of S phase. This will be integral in determining if nickel mediated SLBP depletion is an early pathway of nickel carcinogenesis. Additionally, although both nickel and cadmium have been shown to induce cell cycle changes, neither metal has been shown to decrease S-phase. Previous investigations indicate that nickel induces a slight G2/M block and a very modest increase in S-phase [34]. Similarly, cadmium induced an increase in G2/M and S-phase as well [35]. Given this previous data, it can be concluded that the reduction in SLBP protein is not due to a cell cycle effect but due to a specific effect of nickel or cadmium at the transcriptional/translational level. Finally, it is important to note that nickel, but not cadmium, had very similar effects on H3.1 mRNA polyadenylation albeit, less drastic [21]. This was interesting to us because it indicates that polyadenylation of canonical histone mRNA H3.1 could be a more general mechanism by which carcinogenic metals exert their effects. Although cadmium treatment did not show an increase in poly(A) H3.1 it is possible that other carcinogenic metals might and merits further investigation.

### Possible mechanisms of H3.1 polyadenylation during Nickel Mediated SLBP Depletion

Immature canonical histone pre-mRNAs contain two cis-acting elements that are required for proper processing–the stem loop structure and a TATAA consensus sequence known as the histone downstream elements (HDE). Both elements are located at the 3' end of the mRNA. Trans-acting elements involved in replication histone mRNA processing include SLBP, cleavage factor (CF), heat liable factor (HLF), and the U7 small nuclear RNP (U7 snRNP) [32, 36]. SLBP binds the stem loop structure while U7 snRNP binds the HDE [34, 37]. It is clear that the multi-unit complex cleaves the 3' end of histone mRNA just after the stem loop. The bound SLBP then regulates the trafficking, translation, and degradation of histone mRNA and in the absence of SLBP, histone mRNA cannot be cleaved. Our lab showed that, in the SLBP deficient cell, a downstream poly(A) signal is enriched in histone H3.1 mRNA [22]. Further investigation to determine whether this sequence is enriched in nickel exposed cells would support our hypothesis that inappropriately expressed poly(A) histone mRNA is a direct result of nickel mediated SLBP depletion.

## Conclusion

In closing, this investigation elucidated a novel pathway by which nickel may exert is carcinogenic effects via epigenetic SLBP depletion. We have also shown that nickel mediated SLBP depletion correlates with an increase in histone H3.1 mRNAs with polyA tails and potentially increased mRNA stability and genomic instability as a result. Our original hypothesis that SLBP depletion is mechanism exploited by several epigenetic modulating metals has not been fully supported as cadmium did not have all the consistent changes found with As and Ni and yet other carcinogenic metals (chromium, vanadium, etc.) have yet to be tested. Future experiments should focus on the specific molecular events that facilitate SLBP protein and mRNA depletion. Finally, it would also be interesting to determine the polyadenylation status of other canonical histone mRNAs.
